# Effectiveness of Deep Learning Algorithms to Determine Laterality in Radiographs

**DOI:** 10.1007/s10278-019-00226-y

**Published:** 2019-05-07

**Authors:** Ross W. Filice, Shelby K. Frantz

**Affiliations:** MedStar Georgetown University Hospital, 3800 Reservoir Road, NW CG201, Washington, DC 20007 USA

**Keywords:** Deep learning, Quality, Classification, Object detection, Feedback

## Abstract

Develop a highly accurate deep learning model to reliably classify radiographs by laterality. Digital Imaging and Communications in Medicine (DICOM) data for nine body parts was extracted retrospectively. Laterality was determined directly if encoded properly or inferred using other elements. Curation confirmed categorization and identified inaccurate labels due to human error. Augmentation enriched training data to semi-equilibrate classes. Classification and object detection models were developed on a dedicated workstation and tested on novel images. Receiver operating characteristic (ROC) curves, sensitivity, specificity, and accuracy were calculated. Study-level accuracy was determined and both were compared to human performance. An ensemble model was tested for the rigorous use-case of automatically classifying exams retrospectively. The final classification model identified novel images with an ROC area under the curve (AUC) of 0.999, improving on previous work and comparable to human performance. A similar ROC curve was observed for per-study analysis with AUC of 0.999. The object detection model classified images with accuracy of 99% or greater at both image and study level. Confidence scores allow adjustment of sensitivity and specificity as needed; the ensemble model designed for the highly specific use-case of automatically classifying exams was comparable and arguably better than human performance demonstrating 99% accuracy with 1% of exams unchanged and no incorrect classification. Deep learning models can classify radiographs by laterality with high accuracy and may be applied in a variety of settings that could improve patient safety and radiologist satisfaction. Rigorous use-cases requiring high specificity are achievable.

## Introduction

Machine learning (ML) and deep learning (DL) are artificial intelligence (AI) methods with significant potential to augment both interpretive and non-interpretive radiology workflows. Although clinical applications remain in early development, ML demonstrates capability to influence imaging interpretation and beyond [1–4]. Ongoing research displays promise in diverse applications of diagnosis, enhanced imaging and reconstruction, automated decision support, exam prioritization, and risk prediction [1–6].

ML involves the application of mathematical models to datasets to generate autonomous predictions using new data. Exposure to training data allows the model to learn from errors in processing initial cases with iterative improvement in performance after additional examples. A common ML algorithm is the artificial neural network (ANN) consisting of three segments (input, hidden, and output) of which many hidden layers can exist [1]. DL—an extension of ML—combines trainable units utilizing many layers that can accomplish complex tasks including image classification and object detection [2]. In order to train these networks reliably, large accurately labeled and curated datasets are required [2].

In our practice, we perform approximately 500,000 radiographs per year of which up to half lack properly encoded laterality in the Digital Imaging and Communications in Medicine (DICOM) metadata with a small fraction containing incorrect laterality. When considering laterality-specific body parts, most commonly extremities, absent or incorrect laterality information raises the potential for significant downstream clinical decision-making errors. In a Veterans Health Administration analysis of reported adverse events, 65 out of 210 were wrong-side procedures [7]; such adverse events could plausibly arise from incorrectly labeled radiology examinations used for planning or decision-making.

Missing laterality data also poses quality and workflow challenges. Hanging protocols in Picture Archiving and Communication Systems based on DICOM metadata typically use laterality tags; if this tag is missing or incorrect, a relevant prior may not be shown, or worse, a prior contralateral body part may be shown. This creates situations where a radiologist may, at best, render a suboptimal interpretation due to absent data or, at worst, render an inaccurate interpretation from inappropriate comparison to a prior of opposite laterality.

Previous work has shown, as a secondary outcome, that there is promise for DL models to classify radiographs by laterality [8] and tangentially that laterality markers can be detected by classification models [9, 10]. However, reported accuracy rates were lower than desired for actual clinical application. We sought to build on this work and improve classification accuracy to a level comparable to human performance such that it could be used clinically for both quality control and retrospective archive correction.

## Materials and Methods

Institutional review board exemption was obtained. Data was acquired from January through July, 2018, and was handled in HIPAA-compliant fashion.

### Image Dataset Acquisition and Curation

We randomly queried operational databases for a wide variety of laterality-specific radiographs. Accession numbers were hashed to ensure anonymity with a secure lookup table retained for traceability. Pixel data was extracted, duplicate images were removed, and laterality information was determined directly from DICOM metadata (0020,0060) or inferred based on other study information such as study or series description. Distinct naïve datasets were set aside for testing. No images were excluded. A dedicated workstation with a high-end graphical processing unit (GPU) was utilized.

All datasets were manually reviewed by a fourth-year medical student [SKF] and again by the supervising attending radiologist (9 years experience) [RWF] to ensure correct categorization by consensus. Lead markers within the images were considered ground truth; when errors were found, images were moved to the appropriate category. Images with missing or uninterpretable lead markers were placed in a third “unknown” category as any model considered for real-world use must be able to identify such exams. A more detailed review was performed on 4357 random representative images to establish baseline technologist error rate.

We ultimately produced a training set of nine distinct laterality-specific body parts [Table 1]. Through curation, we generated a third “unknown” category of 237 unique images. In an attempt to better equilibrate our training data, we augmented the “unknown” images by 90° rotation and flipping as lead markers are not infrequently reversed or rotated. Our final classification dataset included 9437 training images with 3146 validation images and 2822 images reserved for testing. Images for classification were rescaled and interpolated to fill a 256 × 256 pixel matrix to match existing pretrained networks. A peripheral black border of 26 pixels was introduced based on early anecdotal experience suggesting improved performance for markers found frequently at the edge of images.

**Table 1:** The nine body parts used for development of the deep learning models

Body parts	
Ankle	
Elbow	
Femur	
Forearm	
Hand	
Humerus	
Knee	
Shoulder	
Tibia/fibula	

In total, 1273 images were randomly selected to develop an object detection model. Bounding boxes were drawn around the lead markers by the supervising attending radiologist. The original images were rescaled and interpolated to a 1200 × 1200 matrix to facilitate more reliable object detection. One thousand eighteen images were used for training with 255 for validation and a novel set of 292 images from 50 left and 50 right examinations was reserved for testing.

### Model Design and Refinement

We developed classification models using the pretrained GoogLeNet [11] or AlexNet [12] networks. Multiple variables were experimented with including solver/optimization algorithm type, number of training epochs, base learning rate, learning rate decay, batch size, and image mean subtraction. Optimization of the algorithm was performed based on increasing validation accuracy while avoiding overfitting (i.e., training loss substantially less than validation loss). Our object detection model was developed based on an extension of the BVLC GoogLeNet [13] network called DetectNet [14] with further modification of the clustering layers to allow simultaneous detection of two different objects (L and R lead markers).

### Evaluation of Model Performance

Our classification model returned a prediction for each class, “R,” “L,” or “U,” with a confidence score based on the final softmax layer probability. The object detection model returned coordinates for detected objects along with confidence scores.

Study-level classification accuracy was determined using two methods:

1. We assigned laterality to a study based on majority rule. For example in a three-view study, if our model proposed “R” for two images and “L” for one, we assigned “R” study-level laterality. If there was a tie in two-view or four-view radiographs, we assigned laterality using the highest average confidence score from each class.
2. We used confidence scores for “L” laterality and multiplied − 1 by the confidence scores for “R” laterality. We then took the mean of all confidence scores with a positive mean corresponding to “L” and negative mean to “R.”

An ensemble method was considered in an attempt to improve performance in particular for the rigorous use-case of automatically classifying historical data. A screening confidence threshold for the classification model was chosen based on the ROC curve correlating with a true positive fraction of at least 99%. Any images below this threshold were classified using the object detection model with confidence scores used to break ties in the infrequent case of multiple detected objects. Study-level laterality was then determined using majority rule based on ensemble image classification. If majority rule could not be determined, the study would be left unaltered.

Confusion matrices were produced to assess classification performance. A web-based program based on the JLABROC4 library [15] for continuous data was used to generate receiver operating characteristic (ROC) curves based on confidence scores which facilitated adjustment of sensitivity and specificity as appropriate. Assessment of object detection was performed by manual validation with overall sensitivity, specificity, and accuracy determined for individual markers as well as for each exam.

## Results

### Data Curation

The 15,405 unique images curated for training, validation, and testing came from 4619 unique exams, of which 694 (15%) were missing DICOM laterality data in all included series and images. Sixty-seven images from 23 exams had frankly incorrect DICOM laterality data compared to the lead marker. Two hundred thirty-seven images (1.5%) had insufficient image markers; these then comprised our “unknown” dataset. All images, regardless of presence or absence of DICOM laterality tag, were reviewed manually by consensus [SKF, RWF] to ensure accuracy.

### Model Design and Refinement

**Fig. 1:**
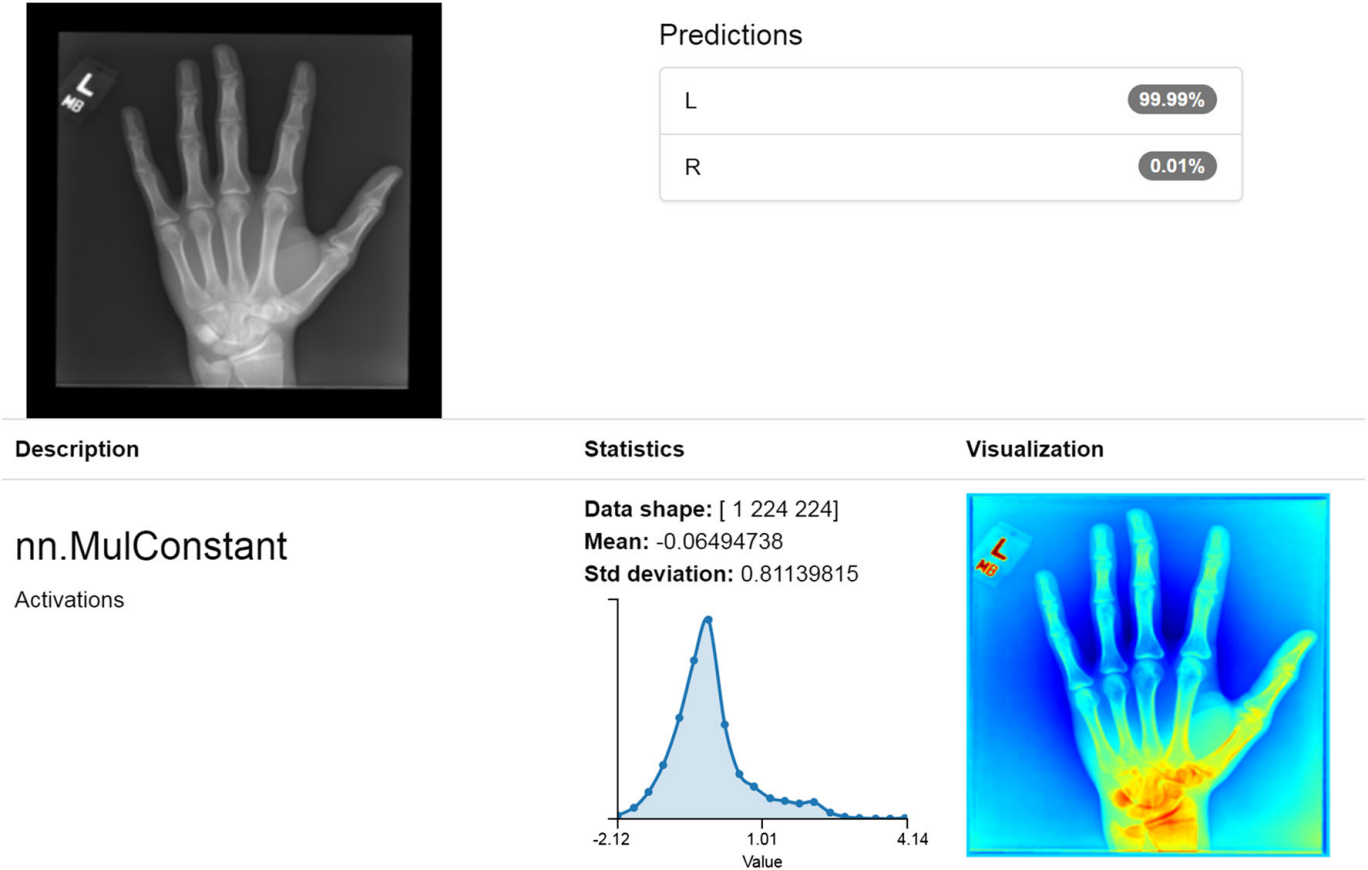
Correct classification with 99.99% confidence in left hand radiograph. Image depicts the model’s results of classification task on a single left hand radiograph, revealing 99.99% confidence in an accurate left laterality prediction. Convolution metadata appears to correctly identify the lead marker as the salient classification feature

For the classification model, we found that using the Torch framework starting with the GoogLeNet pretrained network performed best with mean image subtraction and Nesterov’s accelerated gradient for loss optimization with a base learning rate of 0.01 and polynomial decay using a power of 3.0. Loss quickly decreased with only 15–20 epochs required to achieve high accuracy before signs of overfitting were observed. Based on convolution mapping metadata, our models appeared to successfully, and perhaps not unexpectedly, identify the lead markers as the critical classification features [Fig. 1].

For object detection, we found the modified DetectNet network performed best using adaptive moment estimation for loss optimization with a base learning rate of 0.0001 and exponential decay using a gamma of 0.98. Mean average precision quickly rose after approximately 15–20 epochs with further refinement and improvement seen out to 160–200 epochs without signs of overfitting.

### Evaluation of Model Performance

**Fig. 2:**
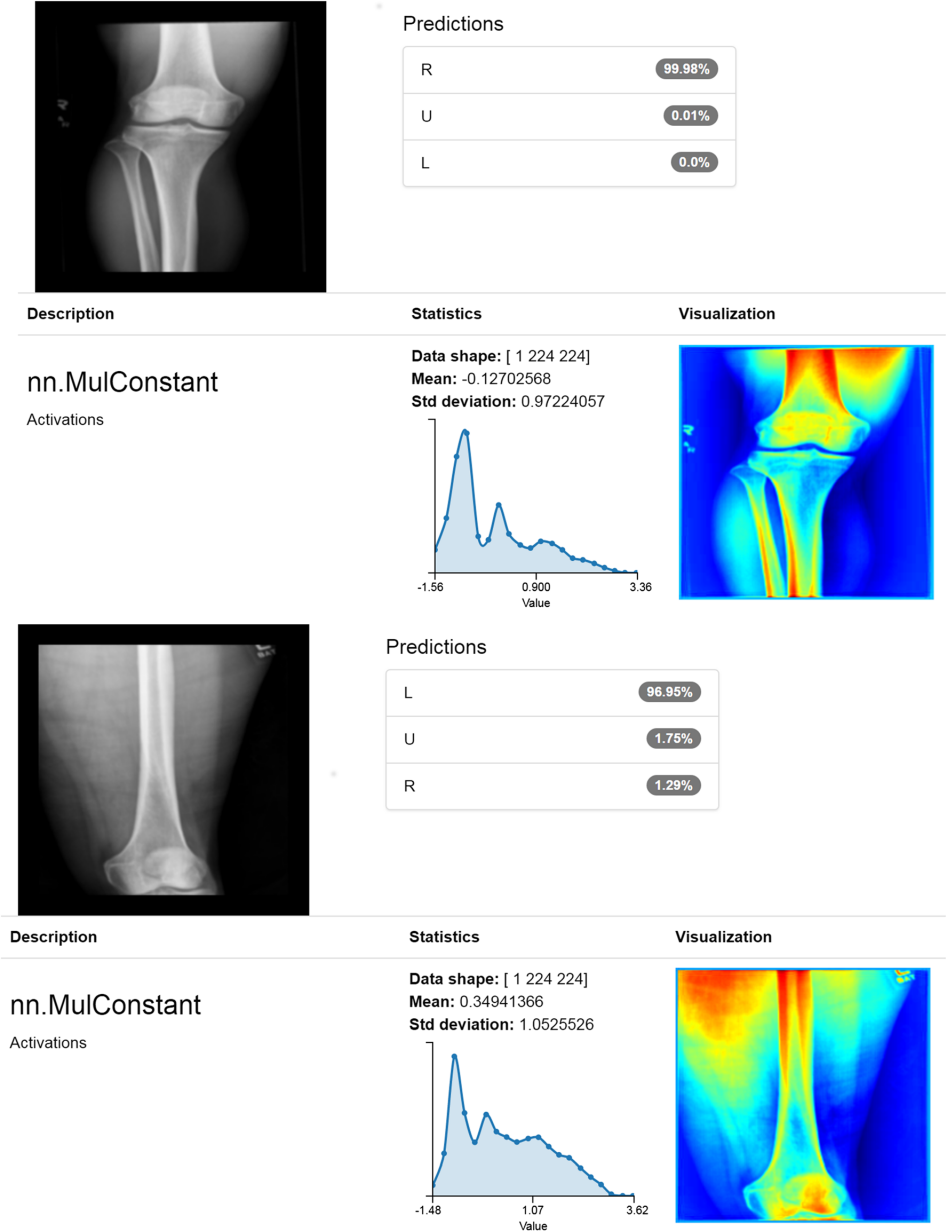
Correct classification of “unknown” partially visible markers. Manual curation established “unknown” ground truth for missing or partially visible markers that were thought to be non-interpretable. Unexpectedly, the model correctly classified some right (top) and left (bottom) “unknowns” due to presence of just enough of the lead marker to make a determination

We then assessed overall performance of our classification model. Two test images placed initially into our “unknown” category due to what we believed were partially visible markers were correctly classified as “R” or “L” with very high confidence. While unanticipated, this result highlights the ability of the algorithm to determine laterality with high accuracy, even in instances of only partially visible lead markers [Fig. 2]. The overall confusion matrix for our classification model demonstrated high accuracy in particular for left and right images [Table 2].

**Table 2:** Confusion matrix for final classification model including per-class accuracy

		Ground truth class			
Predicted class		L	R	U	Per-class accuracy
	L	1383	14	22	97.46%
	R	6	1341	10	98.82%
	U	3	5	38	82.61%

*L*, left; *R*, right; *U*, unknown

**Fig. 3:**
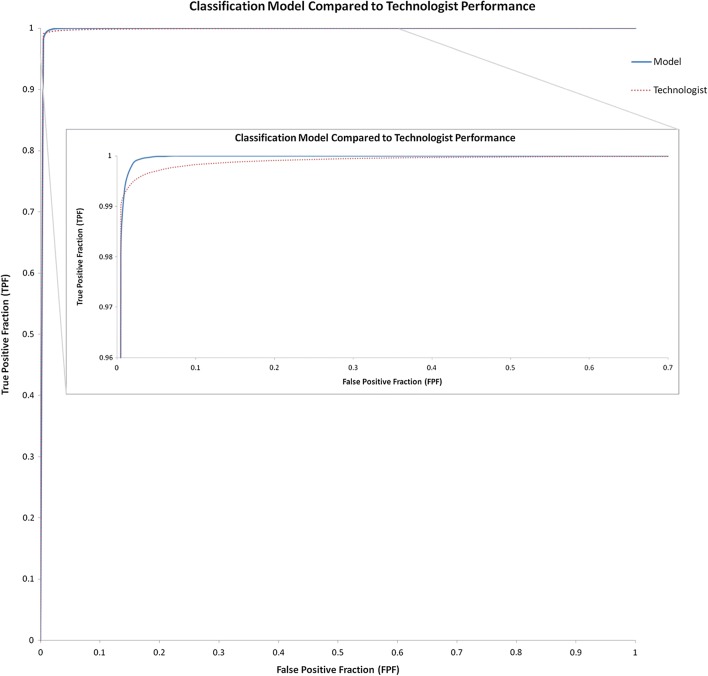
Classification model compared to technologist performance. Classification model and human performance AUC was the same at 0.999 but at the upper left of the AUC, it appears that while humans slightly outperform the model at the lowest false-positive fraction, the model reaches a higher true positive fraction sooner and more consistently

**Fig. 4:**
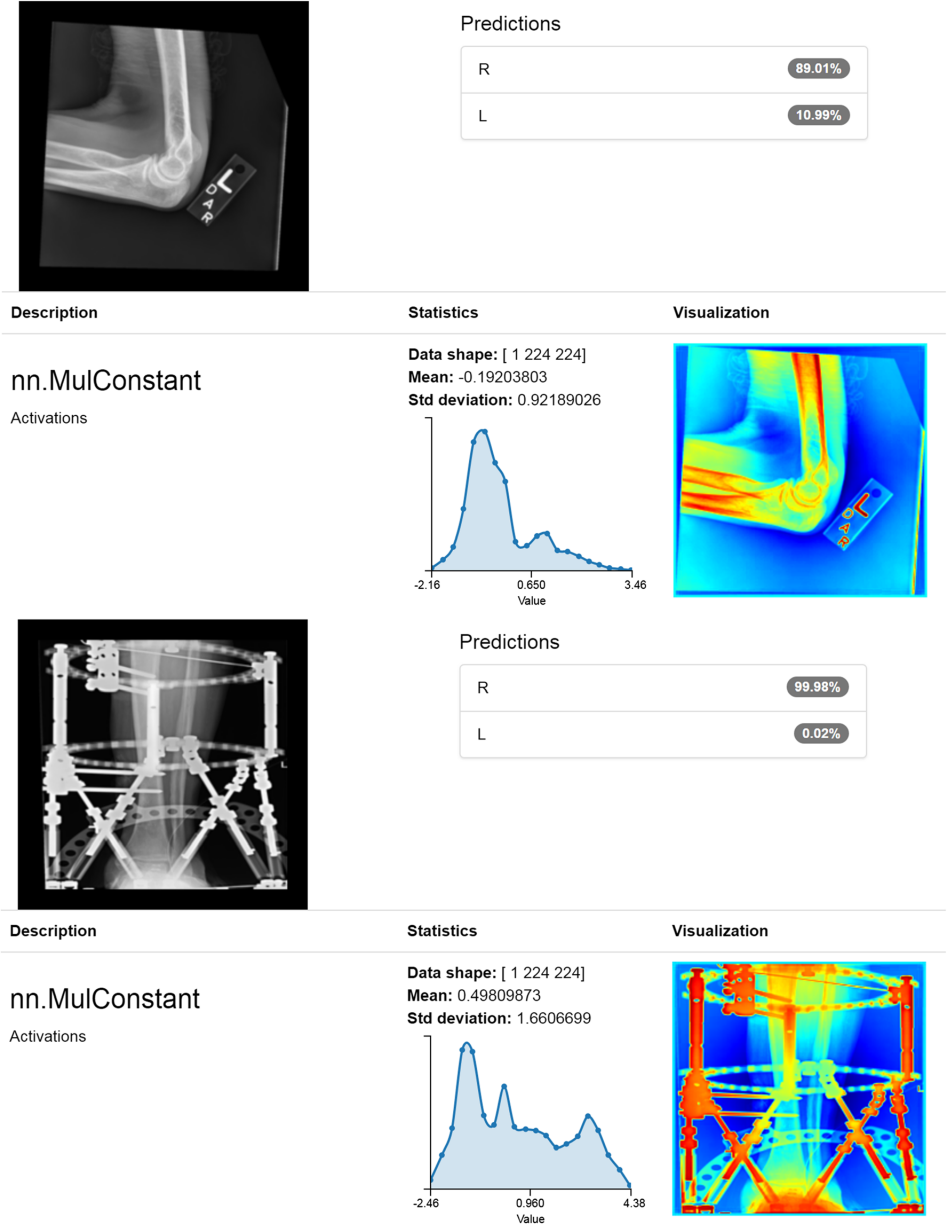
Incorrect classifications. The top image depicts a case when technologist initials were on the lead marker. We propose that initials of “R” or “L” may confound the model. The bottom image depicts an incorrect right classification likely due to extensive amounts of hardware with similar density to lead markers which confounds the model

A multi-class ROC curve for the classification model was also generated by performing a pairwise comparison of the confidence scores for the two classes of interest, “R” and “L,” resulting in an AUC of 0.999 which was comparable to real-world human performance based on our observation of labeling errors in our technologist curated dataset revealing the same AUC of 0.999 [Fig. 3]. Upon review of the few incorrect predictions by our model, we believe many were explainable [Fig. 4] and might be addressed by further generalization or incorporating object detection or ensemble methods. Of note, the eight “unknown” cases that were incorrectly categorized had confidence scores below 83%; if this were applied in a use-case requiring high specificity such as our proposed ensemble model, the confidence threshold would exclude these incorrectly categorized unknowns. Some classifications for long bone exams had lower confidence scores, though still correct, that we believe may be related to our interpolation preprocessing step. Finally, our classification model performed similarly and perhaps slightly better than humans suggesting automated applicability for high volume relabeling or curation.

While our model performed well on a per-image basis, we were also interested in study-level performance. The first analytic method, described above, resulted in the best study-level performance with an ROC AUC of 0.999 but the second was comparable with AUC of 0.998. Therefore, study-level analyses and per-image performance were near equivalent.

**Fig. 5:**
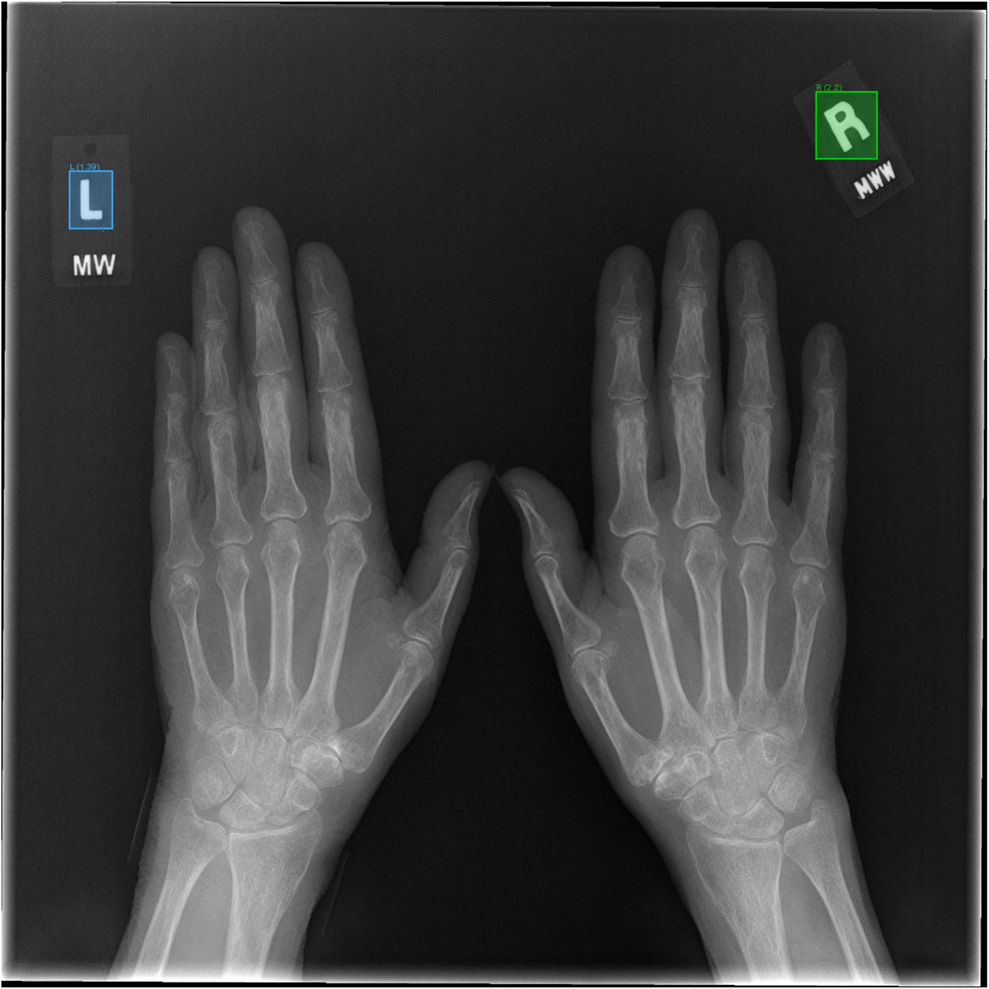
Simultaneous detection of right and left laterality lead markers. The object detection model reliably and simultaneously detects both right and left lead laterality markers with comparable performance to the classification model and offers additional assurance in an ensemble model

For our object detection model test set, 290 of 292 test images contained valid lead laterality makers. Two images were considered negative because of missing or marginally visible markers. Markers were detected simultaneously and in some cases were both on a single image [Fig. 5]. One hundred forty-three true positive left markers were identified with 144 true negatives, 1 false positive, and 0 false negatives for 100% sensitivity, 99.3% specificity, and 99.7% accuracy. One hundred forty-six true positive right markers were identified with 144 true negatives, 0 false positives, and 1 false negative for 99.3% sensitivity, 100% specificity, and 99.7% accuracy. On an exam level, all 50 left exams were categorized correctly for 100% sensitivity, specificity, and accuracy. Forty-six right exams were categorized correctly, 3 were categorized correctly by majority rule, and 1 was split (1 true positive, 1 false negative) for 98% sensitivity, 100% specificity, and 99% accuracy [Table 3]. Long bone radiographs did not appear to be affected as in our classification model, perhaps because of the larger interpolation matrix.

**Table 3:** Object detection model performance assessed by sensitivity, specificity, and performance on an image and study level

	Object detection performance		
	Sensitivity	Specificity	Accuracy
L (image)	100%	99.3%	99.7%
R (image)	99.3%	100%	99.7%
L (study)	100%	100%	100%
R (study)	98%	100%	99%

*L*, left; *R*, right

For the ensemble method, we randomly selected a naïve set of 100 studies of variable laterality-specific bone radiographs consisting of 312 images. Image-level accuracy was 97.8% (305/312) alone and 98.4% (307/312) with the addition of confidence scores from the object detection model to break ties in cases where multiple objects were detected. Study-level accuracy was 99% (99/100) with one study left unclassified because it only contained two images, one of which was classified correctly and the other classified as indeterminate by both models. Importantly, none of these studies would have been retrospectively classified incorrectly but rather the single indeterminate study would have been left alone resulting in substantial improvement in historical radiograph laterality classification without error.

This not only improves on previous work [8] but is also comparable to human-level error; our technologist curated dataset above demonstrated 69/4357 mislabeled or unclear images for image-level accuracy of 98.4% and 3 left and 4 right exams out of 1483 were incorrectly labeled by technologists using majority rule for study-level accuracy of 99.5%. While technologist study-level accuracy is slightly higher overall, the incorrect exams were explicitly wrong whereas our ensemble methodology is highly specific and would not alter data incorrectly.

## Discussion

We developed two robust and highly accurate deep learning models that accurately categorize radiographs by laterality including explicitly identifying images with missing or insufficient lead markers. Confidence scores are generated which can be used to adjust sensitivity and specificity for a variety of real-world use-cases. When combined in ensemble fashion, we believe these methods are highly reliable to use both for automated retrospective data population and for quality assurance at the time of exam.

When compared to human performance, our model performance proved at least equivalent if not better in that errors are not introduced when used in ensemble fashion. We believe this demonstrates that they could be used to retrospectively encode the large number of exams without DICOM encoded laterality at our institution automatically. For this task, our highly specific ensemble methodology would be utilized to ensure acceptable specificity while maintaining adequate sensitivity to result in few inaccurately labeled or unlabeled exams, particularly if all images in a study are considered in context. Overall, we believe such performance would be preferable to the current state with substantial numbers of radiograph images lacking proper programmatic DICOM encoded laterality. Hanging protocols, in particular inaccurate relevant prior selection which we find to be a frequent complaint, would be improved.

Since we can process individual images in a few seconds, these models could be deployed to process images on or even prior to archive ingestion and before interpretation by the radiologist with notifications to the technologist in cases of possible labeling error or unlabeled data. In this case, sensitivity could be increased at the expense of specificity while still maintaining high accuracy, as one would likely be willing to tolerate a few false-positive notifications to ensure accurate placement of lead markers and prospective encoding of DICOM laterality metadata. It has been well demonstrated in similar cases such as flagging report errors or providing feedback on exam duration that analogous continuous quality assurance feedback results in consistent error correction and lower baseline error rates. Immediate and consistent feedback applications hold the potential to improve awareness and baseline functioning and are essential in a field where error must be minimized [16, 17].

### Limitations and Future Directions

We have discussed errors above where it appears that technologist initials or extensive amounts of hardware may confound our models. Improved performance may be achieved by training our model with still more generalized data including a heterogeneous set of similar confounding examples, but this would require additional time-consuming manual effort. This process could perhaps be expedited with the assistance of natural language processing or other semi-intelligent text mining techniques.

We also found that our preprocessing transformation steps of interpolation may not work as well with original pixel matrices that are not near-square such as long bone exams, though performance was still excellent for both models in this study. Different preprocessing steps without interpolation may help our models or further iterations perform even better. Additional generalization could increase the robustness of both models; this might include rare body parts or parts without laterality that still contain lead laterality markers (i.e., chest and abdomen radiographs). Additionally, developing and testing our model and its future iterations across multiple institutions could further demonstrate generalizability. Other deep learning networks or approaches such as segmentation could be explored to improve performance and see if other useful information can be extracted.

While we believe we have improved on previous performance and have achieved accuracy comparable to human performance, it could be useful to develop a public dataset for different research groups to compare to or compete against. An interesting future direction would be either publishing our internal dataset or labeling a currently available public radiograph dataset for this purpose to allow such comparison.

## Conclusions

Deep learning models can be used to classify radiographs by laterality, including an unknown category where markers are missing or uninterpretable, with very high accuracy. Because confidence scores are generated, these models can be deployed in a number of settings with parameters adjusted for desired sensitivity and specificity that could improve both historical and prospective incoming data to improve patient safety and radiologist satisfaction. Future research could target enhanced generalizability across a wide variety of studies and institutions, explore other methods of preprocessing data, and evaluate other deep learning methodologies for potential performance improvements and other important feature extraction.
